# On the Difficulties of the Study of Prevailing Diseases

**Published:** 1858-10

**Authors:** W. H. Michael

**Affiliations:** Mayor of Swansea.


### ON THE DIFFICULTIES OF THE STUDY OF PREVAILING DISEASES.

By W. H. MICHAEL, Esq., Mayor of Swansea.

[*Read before the Society on Monday, July 5th,* 1856.]

THESE two questions require solution.

1. What are the causes which determine the advent of disease, in such large proportion to the population of any district, as to justify the title of epidemic visitation; and

2. What causes operate to check the progress of an epidemic, to moderate its diffusion, and to bring about its departure?

Are these causes to be sought in the air we breathe, or in the food and water we ingest? Do they depend on some subtle electrical phenomena, which depress vital agency and make the body prone to take on disease, or is there some telluric influence which re-acts in a particular manner, according to some definite but uncomprehended general laws, predisposing to a particular disease, or a group of diseases; for experience amply proves that such diseases as smallpox and measles, hooping-cough and scarlet-fever, may not only exist together in the same district or town, but may also be present in the same household and family, or may even coexist at the same time in one individual. Has relative height of locality any influence in procuring exemption from pestilence; and, if so, how are the exceptions to this explained away, and what are the general laws which influence the velocity of transmission of disease to various parts of a district. Can the germs of disease be communicated by food or water from person to person? Is the contagiousness of disease an accidental circumstance, arising from the intensity of the poison, if such exist? Are all diseases of an epidemic type more or less, under differing circumstances, communicable by contact or infection, as separated from some generally prevailing cause? Are foul smells in themselves capable of producing disease; or is there of necessity before such effects are seen, the presence, undetected by the senses, of some influence or emanation, separable from and independent of them? And is it not possible to deodorize, and yet not disinfect any supposed centre or cause of disease?

These, and many other important questions, have not yet received that attention and that amount of research of an inductive character, which alone can solve definitively these great problems of life and death. This is for the future. In the present, it is well for us rightly to estimate the difficulties in the way of progress, to make sure the ground we have gained, and from the histories of epidemics already possessed to endeavour to deduce those great general laws, without the knowledge of which we can never adequately be masters in epidemiology.

Can we say more than this of the knowledge we have hitherto acquired, when carefully separated from loose generalization, reckless hypothesis, and unsupported theory,—"That whatever tends to depress nervous and muscular power and energy, acts as a promoter of cholera, smallpox, fever, and allied disease:—That, therefore, those are most prone to these attacks, who live in ill-drained, badly ventilated houses, crowded together in dirty close rooms, in back streets or alleys of towns, where there is no free passage of air:—Where hard labour and wasting fatigue can only procure a scanty pittance for the support of the family, or where even this is squandered in intemperance:—That exposure to cold and wet, to watching, anxiety, or fear; that these and all other causes of a similarly prostative character prepare the way for surrender to prevailing morbid influences"?—But what are these, and why are they present only at particular junctures, all surrounding circumstances being apparently the same? And what, if any, is the pabulum which feeds cholera in distinction to typhus, and entails on a community hooping-cough or smallpox? It is much easier to place on record these difficulties, than to afford any sufficient answer. It is hardly increasing our knowledge to say, "That disease begins where resistance ends," or that visitations of disease leave a locality where they have weeded out from the inhabitants those ready to take on any disease, and that having fed on the weak, the drunken, and the destitute, starved out, they seek some other more congenial locality. The first of these, in sonorous language, covers our ignorance; and the second is being proved to be untrue by the recurrence of attacks of epidemic disease, when apparently they had entirely ceased, only differing slightly in their type or the class of persons visited by their attacks. It is a grave matter for consideration, why, if local causes are competent to produce zymotic diseases, are they not always present, the surrounding circumstances being the same; and if the germs are latent, what is it that leads to their development into fatal vitality and death-bearing power? Is it not possible that we may by inquiry find that, (like yellow fever), other diseases may be localized, and the idea of their contagiousness disproved?

In this case, experience has shown us that by exposing a body of men to certain influences, we inevitably produce a disease; and by removal from these surrounding causes, may destroy its malignity, and at last ensure its absence. Nay, more, that we can carry about the disease by transporting with our men African mud in the hold of our vessels, and so make the disease assume the three types of epidemic, endemic, and contagious.

We may come home, however, for an illustration, and ask some questions of the notorious night-soil fever, so prevalent under certain atmospheric conditions in towns, where the cess-pit system extensively prevails. Here we may see a disease of the epidemic class clearly traceable to local causes, and capable of being removed by the appliances of art. Its positive symptoms and typhoid characteristics, partaking especially of the marks of blood-poisoning. A gentleman, in robust health, superintending the cleaning out of a privy and cess-pit attached to a public market, was suddenly seized with vomiting. He had, subsequently, rigors, loss of appetite, great prostration and malaise, and had a bad taste in his mouth for four or five days. Here was a case of poisoning from foul and offensive smell, or the poison conjoined therewith. But to each house in country towns, and, unfortunately, most often close to each house, is attached such a privy and cess-pit, very generally unprotected; with its more fluid contents allowed to drain off into the adjacent ground, until it becomes saturated with the offensive and unwholesome matter. Why is it that, if such emanations, as in the case I have recorded, are liable to be given off, and almost continually are so, that the families who live in the adjacent houses are not more constantly affected with the class of diseases these emanations are proven to be capable of producing. I believe that it is to the fact of earth being our most sure and safe deodorant and disinfectant, that we must attribute the immunity.

But the soil becomes surcharged, and often in the dwellings themselves, gives off noxious emanations. It may be in such intensity as to give rise to fever, to diarrhœa, or other symptoms of blood damaging; but more often, in so gradual a manner is there a vitiation of the air respired, that the health of the inmates becomes insensibly worn down, and they become ready to receive any disease prevailing. Can we thus account for hooping-cough, croup, and allied diseases, being so rife in some crowded and ill-drained localities?

Drainage, properly carried out, will undoubtedly much diminish suffering and mortality from this cause; there are however special dangers to be guarded against and avoided, lest we bring on families, by escape of the pent up gases in ill-regulated sewers, most direful visitations of disease. Does not a reference to this fact explain the prevalence of typhus fever in elevated country spots, where the air in the immediate locality of the houses is loaded with offensive smells from every kind of impurity, stored up or allowed to find a natural channel: the field or garden being the usual receptacle for the refuse of the family. But experience seems to point to another mode in which the polluting influence of the cess-pit system is felt. It most powerfully reacts on the public health, by poisoning wells of water most generally placed in direct approximation to the unprotected cess-pit walls. Can it, however, be proved that this is ever the direct agent in the propagation of cholera and like diseases? If so, how does the first case occur often in persons who have never previous to their attack left their own habitation, or come within the sphere of the morbific agency of air or water, contact, or infection.

Why is it, if this be the *modus agendi*, that the first cases of any epidemic are almost invariably the most severe and fatal, and why do they occur in districts in which no communication has taken place with infected localities. Can we then solve this difficulty, how are epidemics generated? or how do the first cases really epidemic arrive? Scarlatina has not existed in a given locality for six or eight months, or cases have occurred at long distances apart in a town, when, suddenly, there are cases in almost every house. Has the cause of this sudden outbreak originated from agents always present, or has it travelled from a distance?

Smallpox travels over the principality of Wales, in succession visiting every town and almost every hamlet for six months. In Swansea, isolated cases from infected districts appeared in the autumn of last year, but no other persons were attacked than those brought into actual contact, and those only in very few instances and at long intervals. The disease appeared to have vanished when, suddenly, as in a day, there were dozens of attacks in an intense form; and the visitation went on until it appeared to have attacked all liable to its influence. Of this gradual approach and decline, the last epidemic affords conclusive statistics. In 1849, no fatal case occurred. In 1850, one fatal case. In 1851, two fatal cases. In 1852, one hundred and four fatal cases. In 1853, ten fatal cases. In 1854, no case.

These are the records of the disease in the town of Swansea, and undoubtedly find confirmation in the history of other towns similarly situated. It has usually been believed, that epidemics cease when all who are liable to them have been attacked; but the histories of late epidemics of small-pox, show that after apparently declining and passing on to another locality, they have returned after a month's absence, in a somewhat altered type and attacking a differing class of persons. How can this be accounted for by our present knowledge? We require a carefully recorded register of observations of the time of advent of all epidemics in districts adjoining each other, accurately prepared records, on an uniform plan by competent observers of the atmospheric and other phenomena which preceded, accompanied, or followed them. We want to know more of the influence exerted by geological formation, in determining the severity of visitations of disease, and the time they linger on the site, and their rate of travel over different conditions of soil, elevation, and density of population.

Why is it that different visitations of the same disease are felt upon successive eras in differing parts of the same town or district, defying elevation, and the ordinary laws of sanitary exemption? Can one difference, such as supply of water, be sufficient to control all other causes? In 1832, the borough of Swansea, almost entirely undrained, was first visited by cholera. The disease prevailed largely in the low lying parts of the town. In 1849, that portion was supplied with water brought from a distance, and almost entirely escaped, the limit of the disease being almost marked out by the cessation of the water supply; no case, so far as I am aware, occurring in any house where the inhabitants drank this purer pipe water. But in 1854, when, for the third time this disease appeared, hardly a house in the town was visited, but many cases occurred in an outlying part of the borough, three miles from its main streets. In these three cases, the sanitary conditions of the various parts were relatively unaltered, except so far as the water supply in one portion was concerned; and here, it would be thought, looking at its relative elevation, that this would have been a cause sufficient to secure the comparative exemption of the portion actually attacked.

Immediately after the decline of cholera in 1849, a portion of the borough of Swansea, which had only lost six lives from cholera, and the total average mortality from which amounted for five years to forty-one persons of all ages per annum, lost in the three last months of the year, thirty-nine lives from scarlet fever of a fatal and remittent type. This was confined to a most limited area, and for the five succeeding years no single fatal case of that disease has occurred, neither did it prevail beyond that part of the tract, or cross the river to any house on the opposite side.

To what shall we attribute these exemptions, and apparently inexplicable differences in the aspect and locality of disease? Are they the result of fixed laws which it is in our power to analyze and trace; or are they to be considered as beyond our powers of observation and discovery? If the latter, we must cease to call them preventible diseases, and raise the estimate of that mortality which is to be considered inevitable from our present condition.

Why is it that, in scarlet fever especially, but in all epidemic diseases more or less, one house in a row appears to be selected for the full fury of the disease, and this house not the dirtiest, the most overcrowded, or ill-ventilated; in fact, not in any way differing, so far as observation can discover, from other and adjoining houses? And, moreover, can any explanation be given of the fact, that one child not less healthful than the others, shall be so severely visited as to die in a few hours, apparently exhausted by the severity with which the system is invaded by the poison of the disease, while in other members of the family the disease is so slight as almost to escape observation, or only become noted through some of the sequelæ which attend its decline?

Why does the period of infancy pass some in families, or some individuals in families, without any of the diseases commonly believed to be essential to the period of childhood, such as scarlet fever, measles, hooping-cough, and the like? Can the number of those exempted be indefinitely increased by a better understanding of the laws which govern these diseases? As they recur so seldom as to make one attack almost a sure safeguard, may not some means be discovered, as in smallpox, by the substitution of a milder disease, to secure the immunity without incurring the danger? Or are we to believe that these must remain the exceptions, while the attacks of disease continue the rule?

Does not the mere statement of these difficulties, yet unsolved, suggest many others on which information is imperatively demanded? and can we hope to arrive at valuable results, without a staff of observers, whose special duty it should be to protect the public health; and while they should point out the means to avert or lessen the severity of impending epidemics, so to note their attendant circumstances, as to be able eventually to collect and generalize for the use of the nation, facts which are now so loosely garnered and so carelessly stored, as to be almost without value in solving the great problem :—How shall we best neutralize those diseases we believe to be preventible, but which now, unprevented, fatally affect the public health ?

May we not hope by this well directed inquiry, conducted in the true inductive spirit, to be able eventually to discover the latent cause of all zymotic diseases, and to trace to their true sources those scourges of the apathy and crime of mankind, which lie concealed in cholera, smallpox, scarlet fever, and the like ? Great will be the boon to humanity, however ill our labours be requited, if we can add but one step to the ladder which shall reach to that shrine in the temple of truth, where these problems may receive their solution for the world's wellbeing and comparative freedom from suffering, disease, and death.

